# High photobiont diversity in the common European soil crust lichen *Psora decipiens*

**DOI:** 10.1007/s10531-014-0662-1

**Published:** 2014-03-08

**Authors:** Ulrike Ruprecht, Georg Brunauer, Roman Türk

**Affiliations:** Organismic Biology, University of Salzburg, Hellbrunnerstr. 34, 5020 Salzburg, Austria

**Keywords:** Soil crust forming lichens, Genetic diversity, Chlorobiont, *Psora decipiens*, *Trebouxia* sp., *Asterochloris* sp.

## Abstract

The genetic diversity of green algal photobionts (chlorobionts) in soil crust forming lichens was studied as part of the SCIN-project (Soil Crust InterNational). A total of 64 lichen samples were collected from four different sites along latitudinal and altitudinal gradients in Europe (Tabernas/Spain; Hochtor-Großglockner/Austria; Gynge Alvar/Sweden; Ruine Homburg/Germany). The dominant lichen species at all four sites was *Psora decipiens*, often occurring with *Buellia elegans, Fulgensia bracteata, F. fulgens* and *Peltigera rufescens.* Genetic identification of chlorobionts was carried out using the nuclear marker (nrITS) and a chloroplast marker (*psb*L-J). We found *P. decipiens* to be associated with several different species of *Trebouxia* and *Asterochloris*, although previously described to only have *Asterochloris* sp. The phylogenetic analyses revealed a high chlorobiont diversity with 12 well supported clades, including *Trebouxia asymmetrica*, *T. jamesii*, *T. impressa* and other, as yet taxonomically unidentified clades (*Trebouxia* sp. URa1-4, *T*. sp. URa6, *T*. sp. URa7-13). Additionally, five clades of *Asterochloris* were identified (*A. magna, A.* sp. URa14 -17). Most of the chlorobiont species appeared to be cosmopolitan, but five clades were unevenly distributed between the sampling sites with only *Trebouxia* being found in the warm and dry Spanish habitats and combinations of *Trebouxia* and *Asterochloris* in the cooler and more humid habitats. The wide range of chlorobiont species might contribute to the observed domination of *P. decipiens* at all four research sites of the SCIN project which range from a desert in Spain to an alpine site in the Alps of Austria.

**Electronic supplementary material:**

The online version of this article (doi:10.1007/s10531-014-0662-1) contains supplementary material, which is available to authorized users.

## Introduction

**Fig. 1:**
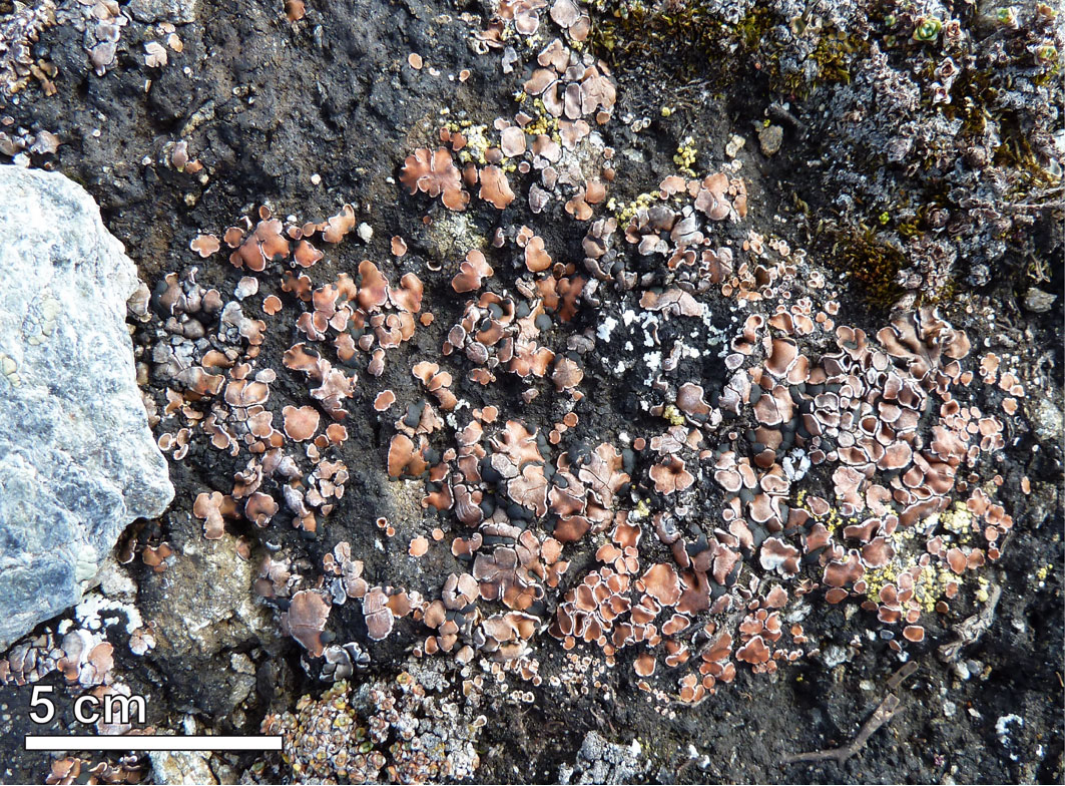
Typical lichen dominated soil crust in high alpine areas, with *Psora decipiens*, *Fulgensia* sp. and mosses

Large parts of the world are covered by soils with a surface vegetative community of lichens, cyanobacteria, micro fungi, algae and bryophytes, so-called biological soil crusts (BCSs, Fig. 1; Belnap et al. 2001). In the absence of larger, higher plants, lichens, small plants and mosses can stabilize the soil surface against erosion and provide shelter to a broad range of insects and other arthropods (Brantley and Shepherd 2004). BSCs also play an important role in the soil water balance and nutrient cycle (Belnap et al. 2001, 2006; Maestre et al. 2011). At first, BSCs were only described for drylands (arid and semiarid areas) which occupy 41 % of Earth’s land area (Adeel et al. 2005), but recently these communities have also been reported in alpine and nival regions (e.g. Türk and Gärtner 2001).

The species composition of BSCs mainly depends on water-availability, climate zone and soil-type (Rosentreter and Belnap 2001). While cyanobacteria dominate soil crusts in hot desert regions, lichens tend to be more abundant in regions with higher precipitation (Belnap et al. 2001). Due to their poikilohydric lifestyle, lichens are very well adapted to extreme habitats with rapid temperature and moisture fluctuations, such as high alpine areas and arid areas with high insolation in southern Europe and other parts of the world (Lange et al. 1997; Lange 2000). BSC-forming lichens are present in different growth forms, crustose, foliose and fruticose, with individual characteristics according to the climate zones (Grube et al. 2010). In particular, crustose lichens like *Buellia* sp. and closely attached foliose lichens, such as the common *Psora* sp., form a compact and stable zone in the upper few millimetres of the substratum (Belnap and Lange 2001). The rhizines and rhizomorphs of lichens can stabilize soils more efficiently than cyanobacterial dominated BSC and contribute to a higher amount of soil carbon and nitrogen, soil moisture and plant-available nutrients (Belnap et al. 2006; Maestre et al. 2011).

Very little is known about either the complexity or basis of the successful strategies of lichens in BSCs despite their world-wide importance (Grube et al. 2010). Because of their slow growth, lichens cannot compete effectively against vascular plants but, in areas with extreme abiotic conditions such as long periods of drought or cold, higher plants are excluded and lichens fill this important niche (Lalley et al. 2006). The symbiotic life form of lichens is composed of a fungal (mycobiont) and an photosynthetic partner (photobionts), and the latter can be an eukaryotic green alga (chlorobiont) and/or a cyanobacterium (cyanobiont). The ability of mycobionts to switch photobionts (Nelsen and Gargas 2009; Otalora et al. 2010; Henskens et al. 2012) and associate with more than one photobiont species or genotype along a climatic gradient appears to be a mechanism used by lichens to adapt to particular habitats. This has been reported for crustose lichens (Blaha et al. 2006; Muggia et al. 2008; Ruprecht et al. 2012) and for fruticose lichens (Kroken and Taylor 2000). The influence of photobiont selection on the ecological amplitude of lichens is still largely underexplored (Peksa and Skaloud 2011) and shedding more light on this phenomenon would help towards understanding structure, composition and development of BCSs (Bowker 2007; Lazaro et al. 2008).

The research reported here is part of the international and interdisciplinary SCIN-Project (Soil Crust InterNational; please see Büdel et al. 2014) which focuses on the biodiversity, the ecological roles and functions of BSCs in four different sites which differ substantially from each other in terms of soil composition, sea-level, seasonal temperatures and precipitation.

An important first step is to identify the photobionts that occur in any particular lichen species. The major goal, therefore, of the present study was determining the biodiversity of green algal photobionts (chlorobionts) of the soil crust lichen *Psora decipiens* by molecular methods. The crustose green-algal lichen *P. decipiens* (Hedw.) Hoffm. [*Lecidea* *d.* (Hedw.) Ach.], to date described as being only associated with *Asterochloris* sp. (Schaper and Ott 2003), is an important component of BSC at all four SCIN locations and provides an opportunity to investigate photobiont heterogeneity within a widespread lichen species. Molecular analysis of the soil crust lichens’ fungal partner is part of another study within the SCIN project. Additionally, we aimed to refine molecular methods to better handle the difficulties that arise in the molecular analysis of soil crust samples because of the presence of multiple organisms.

## Materials and methods

### Investigation sites and material

Sixty-four samples of the key lichen on soil crusts, *P. decipiens* together with other species (see Online Resource 1) were collected at the four investigation sites of the SCIN-Project which cover both latitudinal and altitudinal gradients. For more detailed site descriptions and maps please see Büdel et al. (2014).

1. Tabernas field site, SE Spain (37.0127222°, −002.4356389°). This area is one of the driest regions in Europe influenced by the semi-arid Mediterranean climate. The mean annual temperature with a wide range is 18.5 °C and the mean annual precipitation is 220 mm but highly variable from year to year. The average annual insolation is very high at more than 3,000 h/year. The BCSs cover one third of the area and are dominated by different types of lichens.
2. Hochtor, Großglockner, Alps, Austria (47.0833333°, 012.8500000°). This high elevation site, with an altitude of 2,600 m a.s.l., is influenced by the severe Alpine climate with temperatures around −9 °C in January and 3 °C in July and an annual mean of around −3.0 °C. The annual precipitation is around 2,000 mm/year of which 70 % falls as snow. The BCSc are dominated by lichens together with mainly cyanobacteria and green algae, some bryophytes, and a few vascular plants.
3. Ruine Homburg, Gössenheim, Bavaria, Germany (50.0166667°, 009.8000000°). The climate in this area is warm temperate with an annual mean temperature of 9.2 °C and an annual precipitation of 600 mm. This anthropogenic influenced landscape is covered by a thin vegetation layer (dry grassland) and dominated by cryptogams.
4. Nature Reserve Gynge Alvar, Öland, Sweden (56.5421389°, 016.4783889°). This lowest elevation site is located on the island of Öland situated close to the SE coast of Sweden. With an annual mean precipitation of 450 mm this is the driest area of the whole country. The mean temperature is around 6.5 °C and ranges from −2 °C in February to 17 °C in July. The BSC dominated zones are covered with cyanobacteria, bryophytes and lichens with infrequent higher plants.

## Methods

### DNA-amplification, primer-design, sequencing

Total DNA was extracted from individual thalli by using the DNeasy Plant Mini Kit (Qiagen) according to the manufacturer’s instructions. The PCR mix contained 0.5 units of GoTaq DNA polymerase, 0.2 nM of each of the four dNTPs, 0.3 μM of each primer (0.6 if degenerated) and about 1 ng genomic DNA. The internal transcribed spacer region (ITS) of the photobionts’ nuclear ribosomal DNA (*Trebouxia* sp. and *Asterochloris* sp.) and the chloroplast-encoded intergenic spacer *psb*J-L (*Trebouxia* sp.) were amplified and sequenced with the primers described in Tables 1 and 2. Because of soil crust related contaminations—mainly different eukaryotic algae—highly specific primers were developed for amplifying the target markers from *Trebouxia* sp. and *Asterochloris* sp. The primers psbF and psbR (Werth and Sork 2010) were used to amplify and sequence the *cp*-marker (*psb*L-J for *Trebouxia* sp.) from Antarctic samples that were already known to have *Trebouxia* photobionts (Ruprecht et al. 2012) and from own *Trebouxia* cultures. These sequences were aligned with relevant *cp*-regions of confirmed related *cp*-genomes from Genbank to design more specific primers. To get sufficient PCR-products nested PCR was performed, first with two outer and rather unspecific primers, followed by a nested reaction with the two newly-developed specific internal primers using a touchdown PCR-protocol (see Table 3). For amplifying *Trebouxia* ITS we used the primer pairs 18S-ITS-uni-for and ITS4T for the first PCR and ITS1aT and ITS4bT for the nested reaction. For *Trebouxia* *psb*L-J the primers for the first reaction were psbF and psbR and the nested primers were psbF-sense and psbR-antisense; for *Asterochloris*-ITS amplification nr-SSU-1780-5′ and ITS4 were used for the first reaction and ITS1-sense-A and ITS2-antisense-A for the nested reaction. Several additional algal sequences for *Chloroidium* sp. and several taxonomically unidentified eukaryotic micro algae species were also amplified and sequenced from soil crust samples using primer combinations ITS1T and ITS4T, ITS1T and ITS1aT, ITS1aT and ITS4aT (primer maps and sequences see Tables 1, 2).

**Table 1:** List of primers used to amplify the internal transcribed spacer (ITS) region rRNA and estimated location of primer sites

Primers	Sequence 5′–3′	Temp. (°C)	References
18S-ITS uni-for	gtgaacctgcggaaggatcatt	56.0	Ruprecht et al. (2012)
nr-SSU-1780-5′-mod	tgcggaaggatcattgattc	55.3	Piercey-Normore and Depriest (2001, modified)
ITS1T	ggaaggatcattgaatctatcgt	55.0	Kroken and Taylor (2000)
ITS1aT	atctatcgtgxmmacaccg	54.4	This study
ITS1-sense-A	tccacaccgagmacaac	54.0	This study
ITS2-antisense-A	aaggtttccctgcttgaca	54.5	This study
ITS4	tcctccgcttattgatatgc	55.3	White et al. (1990)
ITS4bT	ccaaaggcgtcctgca	54.3	This study
ITS4aT	atctatcgtgxmmacaccg	54.5	This study
ITS4T	gttcgctcgccgctacta	56.0	Kroken and Taylor (2000)

**Table 2:** List of primers used to amplify the intergenic spacer of the chloroplast–protein of photosystem II (*psb*L-J) and approximate location of priming sites

Primers	Sequence 5′–3′	Temp. (°C)	References
psbR	aaccraatccanayaaacaa	50.1	Werth and Sork (2010)
psbL-sense	ttaattttcgttttagctgttc	50.9	This study
psbJ-antisense	ttcctaaattttttcgtttcaata	50.8	This study
psbF	gtwgtwccagtattrgacat	52.2	Werth and Sork (2010)

**Table 3:** Overview of the multiple conditions used for the various PCR stages

Marker	PCR 1					PCR 2 (touchdown)							
	Primers	Conditions				Primers	Conditions						
								3×	3×	3×		30×	
nITS *Trebouxia*	18S-ITS-uni-for ITS4T	D	95°	00:30	×35	ITS1aT ITS4bT	D	95°	95°	95°	00:30	95°	00:30
		A	56°	00:30			A	56°	55°	54°	00:30	53°	00:20
		E	72°	00:40			E	72°	72°	72°	00:40	72°	00:40
*cp*-*psb*L-J *Trebouxia*	psbF psbR	D	95°	00:30	×35	psbL-sense psbJ-antisense	D	95°	95°	–	00:30	95°	00:30
		A	50°	00:30			A	53°	52°	–	00:30	51°	00:20
		E	72°	00:50			E	72°	72°	–	00:50	72°	00:50
nITS *Asterochloris*	nr-SSU-1780-5′ ITS4	D	95°	00:30	×35	ITS1-sense-A ITS2-antisense-A	D	95°	00:30	×35			
		A	55°	00:40			A	54°	00:30				
		E	72°	00:30			E	72°	00:40				

Every PCR started with an initial denaturation at 95 °C for 2 min

*D* denaturation, *A* annealing, *E* extension

### Phylogenetic analysis

Nuclear ITS sequences were assembled and edited using Geneious Pro 5.3.4 (www.geneious.com) and aligned with ClustalW (Thompson et al. 1994). The alignment was subsequently refined by using the MUSCLE algorithm implemented in the Geneious program and the *psb*L-J-alignment was reworked by hand to correct ambiguously aligned positions. Poorly aligned positions and divergent regions were eliminated from the alignment using Gblocks 0.91b with default settings (Castresana 2000). The congruency of the concatenated *Trebouxia*-alignment was tested by comparing the topology in the single ITS and the concatenated ITS-*psb*F-L trees. Both phylogenies showed similar topologies and the same groups.

Maximum parsimony analyses (MP) were performed using the program PAUP* (Swofford 2003). Heuristic searches with 1,000 random taxon addition replicates were conducted with TBR branch swapping and MulTrees option in operation, equally weighted characters and gaps treated as missing data. Bootstrapping was performed based on 2,000 replicates with random sequence additions. Homoplasy levels were assessed by calculating consistency index (CI), retention index (RI), and rescaled consistency (RC) index from each parsimony search.

Nucleotide substitution models were chosen using JModeltest 2.1.1. (Darriba et al. 2012). The Akaike information criterion selected the GTR model (Rodriguez et al. 1990) + I + Γ (estimation of invariant sites and a discrete gamma distribution) for the *Trebouxia* alignments and TRN model (Tamura and Nei 1993) + Γ for the *Asterochloris* alignment as the optimal models. A maximum likelihood analysis (ML) was performed using the program Garli 0.96 (http://www.nescent.org/wg_garli/Main_Page) with the estimated model (GTR > 6rate, TrN > 010020) and default settings. A nonparametric bootstrap was used to assess robustness of clades, running 2,000 pseudoreplicates.

For Bayesian tree inference a Markov Chain Monte Carlo (MCMC) procedure as implemented in the program MrBayes 3.2. was used (Ronquist and Huelsenbeck 2003). The analyses were performed assuming the general time reversible model of nucleotide substitution including estimation of invariant sites and a discrete gamma distribution with six rate categories (GTR + I + Γ, Rodriguez et al. 1990). A run with 5 million generations starting with a random tree and employing four simultaneous chains was executed. Every 100th tree was saved into a file. Subsequently, the first 25 % of trees were deleted as the “burn in” of the chain. A consensus topology with posterior probabilities for each clade was calculated from the remaining 37,501 trees.

## Results

**Fig. 2:**
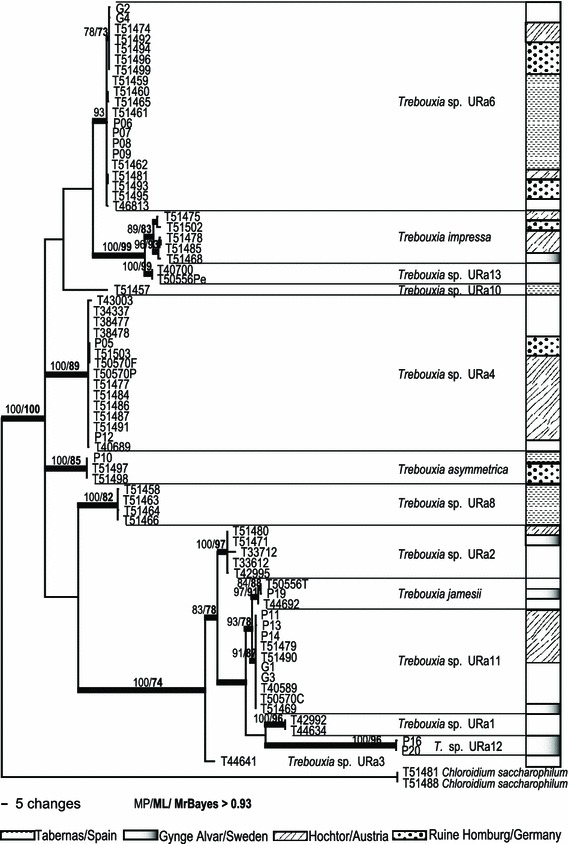
Phylogeny of concatenated ITS and *psb*L-J sequences of *Trebouxia* specimens from the four SCIN-sites, combined with own samples from Antarctica and Austria. The *bars* beside the phylogeny show the provenance of the specimens in the respective habitats. The bootstrap values with >70 support of MP and ML analyses were directly mapped on this Bayesian tree with >0.92 support (branches in *bold*)

**Fig. 3:**
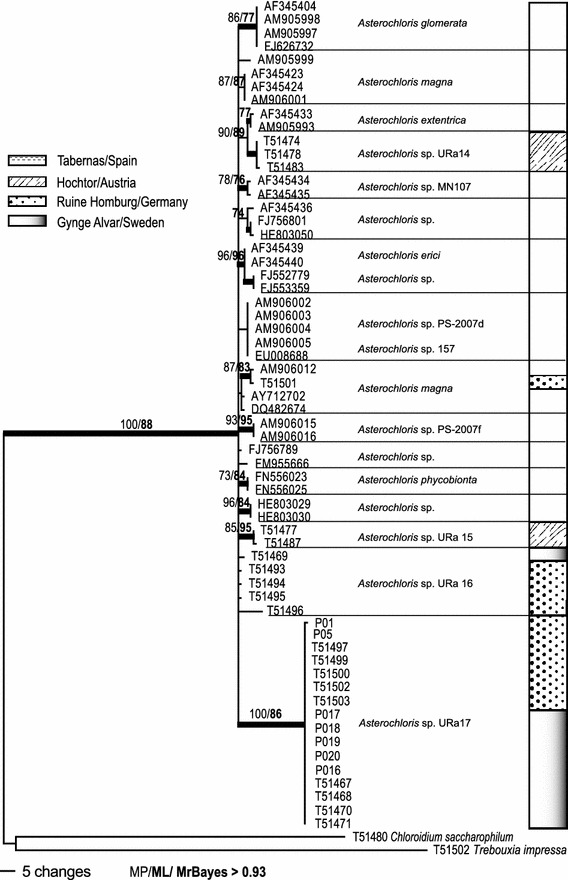
Phylogeny of ITS sequences of *Asterochloris* specimens from the four SCIN-sites, combined with downloaded accessions from Genbank. The *bars* beside the phylogeny show the provenance of the specimens in the respective habitats. The bootstrap values with >70 support of MP and ML analyses were directly mapped on this Bayesian tree with >0.92 support (branches in *bold*)

The final data matrix of the molecular phylogeny of *Trebouxia* ITS (see Online Resource 2) comprised 101 OTUs with a length of 431 characters, 226 positions of the alignment were parsimony-informative with the following homoplasy levels CI = 0.647, RI = 0.953, RC = 0.617. The concatenated *Trebouxia* ITS/*psb*L-J (Fig. 2) phylogeny comprised 75 OTUs with 694 characters, 461 positions were parsimony informative and the homoplasy levels amounted CI = 0.765, RI = 0.958, RC = 0.733. Finally, the *Asterochloris* ITS phylogeny (Fig. 3) comprised 64 OTUs with 435 characters, 169 positions were parsimony informative and the homoplasy levels amounted CI = 0.0 907, RI = 0.943, RC = 0.855. The alignments of *Trebouxia* ITS and *Asterochloris* ITS contained several closely related accessions from Genbank including all taxonomically identified and several taxonomically unidentified species (43 for *Trebouxia*, 35 for *Asterochloris*), plus accessions from other high Alpine and Antarctic areas included in order to get information about intra-specific sequence variation and to see whether the species and haplotypes could be assigned to known clades. Information about the samples is summarized in Online Resource 1.

The ML and Bayesian analyses recovered the same well-supported clades as the MP analysis. The Bayesian consensus trees, with the support values of all three analyses are shown in Online Resource 2 and Figs. 2 and 3. The plotted bars beside the trees show the sample provenance (see also Table 4).

### Phylogenetic analysis

#### *Trebouxia* ITS (Online Resource 2)

This phylogenetic reconstruction was performed to get an overview of the relationship between the photobionts from soil crust lichens and other, already published, sequences of *Trebouxia* species. It revealed 16 well supported, monophyletic groups of which 12 are part of this study and several weakly supported clades of *Trebouxia* photobionts. The tree was rooted with *Chloroidium saccharophilum* the closest related algal group. In addition to the already well known *Trebouxia* species (*T. showmanii*, *T. gigantea*, *T. asymmetrica*, *T. arboricola*, *T. decolorans*, *T. jamesii*, *T. impressa*) and other published but taxonomically unidentified clades (*T*. sp. URa1-4, *T*. sp. URa6 resp. *T*. sp. Guzow, etc.), several other clades appeared. The backbone was not well supported and therefore the topology of the different clades to each other will not be discussed.

A new and well-supported group with four accessions occurred only in Tabernas and was closely related to *T. gigantea*.*T. asymmetrica*, which contained two accessions from Ruine Homburg, was a sister to clade *T*. sp. URa4 found in several accessions from Hochtor as well as from Ruine Homburg.

Another new group (*T.* sp. URa11), formed by several accessions from Hochtor and Gynge Alvar, was sister to *T. jamesii* and to the endemic group of Antarctic photobionts found in extremely cold and dry regions (*T*. sp. URa1) as well as to a new and strongly supported clade of two Swedish samples (*T*. sp. URa12).

The heterogeneous clade of *T. impressa* formed a well-supported group and contained samples from Ruine Homburg, Hochtor and Gynge Alvar, together with its strongly supported sister clade of two accessions including two samples which are not from the study areas (high alpine areas in Austria, *T.* sp. URa13).

*Trebouxia* sp. URa6 which included several specimens from Tabernas, Hochtor and Ruine Homburg, was only weakly supported and, finally, *T.* sp. URa2 that frequently occurs in Antarctica was placed together with one accession from Hochtor and one from Gynge Alvar.

#### Concatenated *Trebouxia* ITS and *psb*L-J (Fig. 2)

This phylogeny, including concatenated sequences of nuclear ITS and the intergenic spacer of the chloroplast–protein of photosystem II (*psb*L-J), produced the same groupings as the *Trebouxia* ITS, but they were more strongly supported and better resolved (see *T*. sp. URa2, 4 and 6). The backbone was better structured and several clades clustered clearly together in one well supported subgroup (*T*. sp. URa2, *T.* *jamesii*, *T*. sp. URa11, *T*. sp. URa1, *T*. sp. URa12 and *T*. sp. URa3).

#### *Asterochloris* ITS (Fig. 3)

Finally, the phylogenetic reconstruction of the nuclear ITS of *Asterochloris* samples including several accessions from Genbank showed many low diverged, but well supported and, in the literature described, species (Peksa and Skaloud 2011). The tree was rooted with *C. saccharophilum* and *T. impressa* in order to better see the degree of relationship of the different photobiont groups. The backbone in this phylogeny was not supported. A quite distinct, strongly supported and new clade contained the majority of *Asterochloris* accessions from this study coming from Ruine Homburg and Gynge Alvar. Two other well, and one weakly, supported groups contained the remaining accessions from Ruine Homburg, Hochtor and Gynge Alvar. Only one sample, from Ruine Homburg, clustered together with *A. magna*. No *Asterochloris* sequence was detected from Tabernas.

The summarized phylogenetic results for photobionts showed three delimited algal groups (*Asterochloris*, *Chloroidium* and *Trebouxia*) and several other, but not assignable eukaryotic green micro algae (see Table 4). Five different *Asterochloris* clades occurred in high alpine and temperate regions (Hochtor, Ruine Homburg and Gynge Alvar) but none at the hot and arid Tabernas field site in SE-Spain. Only one species of *Chloroidium* sp. was molecularly identified and occurred at Hochtor. *Trebouxia* was represented by 12 different clades (including two specimens from outside the SCIN-area at Hochtor [*T*. sp. URa13]), and was found to occur in all habitats. Most of the photobionts were cosmopolitan (12 clades) and only a few accessions forming five small groups were restricted to single sample sites (*Asterochloris* sp. URa14, *A.* sp. URa15—Hochtor; *Trebouxia* sp. URa8—abernas; *T.* sp. URa12—Gynge Alvar; *T.* sp. URa13—Hochtor).

**Table 4:** Overview of chlorobiont occurrence in the four SCIN habitats

	Genus	Tabernas/Spain	Hochtor/Austria	Ruine Homburg/ Germany	Gynge/Sweden
Clades/ species	*Asterochloris* sp.	–	2	3	2
	*Chloroidium saccharophilum*	–	1	–	–
	*Trebouxia* sp.	4	5	5	5
	Other EGMA	–	4	7	2

*Other EGMA* other eukaryotic green micro algae

The key lichen *P. decipiens* occurred not only at all SCIN habitats but also in all additional soil crust specimens from other high Alpine areas. In most cases each individual lichen specimen contained one or more photobionts from every clade together with other eukaryotic green micro algae (EGMA; see Online Resource 1).

The species specificity of the mycobiont towards its photobiont was quite low for *P. decipiens*. In contrast, *Fulgensia bracteata* ssp. *deformis* (which has so far only been found in samples from Hochtor) only occurred with *T*. sp. URa4 and *A*. sp. URa15 (the latter until now only known from this area, Figs. 2, 3). *Peltigera rufescens*, known to have a cyanobacterium as its primary photobiont (O’Brien et al. 2005), was also found to be associated with chlorobionts (Henskens et al. 2012). Specimens of *P. rufescens* from Ruine Homburg were associated with *T.* sp. URa6 and *A.* sp. URa16, although other chlorobionts were available at the site; at Hochtor *P. rufescens* was found with *T. impressa* (see Online Resource 1, Figs. 2, 3).

## Discussion

This evaluation of European lichen-dominated soil crusts from four geographically and climatically diverse sites revealed an unexpectedly high diversity of photobionts in association with the dominant lichen *P. decipiens*. Until now, only the genus *Asterochloris* has been described as the photobiont of *P. decipiens* (Schaper and Ott 2003), but we detected 12 different groups of the genus *Trebouxia* as well as other eukaryotic green micro algae like *C. saccharophilum*. Several of these micro algae are already known to exist as lichen photobionts, such as *T. impressa*, *T. asymmetrica* or the, as yet undescribed, *Trebouxia* sp. URa2, URa4, URa6. The latter three species have also been identified as photobionts from crustose lichens (Ruprecht et al. 2012). Other *Trebouxia* species that are known as free-living algae (e.g. *T. arboricola*; Ettl and Gärtner 1995) were included in the analysis but not found in the soil-crust samples. *P. decipiens* at Hochtor showed a shared use of the available photobionts with other lichen species that were present (see Online Resource 1) with each species having a different level of specificity towards to its photobiont. We can conclude for *P. decipiens* that this lichen is not limited to a single species or even genus of photobiont but instead associates with a broad range of apparently locally available algae. The low specificity of *P. decipiens* for its photobiont might contribute to the broad ecological amplitude of this lichen, a possibility already described for other lichens (Blaha et al. 2006).

Most photobiont species, especially from the genus *Trebouxia,* are cosmopolitan with more or less broad ecological preferences (Fernandez-Mendoza et al. 2011; Ruprecht et al. 2012) and this was true for the most commonly detected clades in this study. However, several distinct and strongly supported clades of the genera *Asterochloris* and *Trebouxia* (Online Resource 2, Figs. 2, 3) do not seem to be cosmopolitan, e.g. *T*. sp URa8 which, to date, has only been found at Tabernas. This clade is sister to *T. gigantea*, a photobiont which is widely distributed in temperate habitats (Ettl and Gärtner 1995). This is a somewhat similar situation to that found in another study of the cosmopolitan photobiont *T. jamesii*. Ruprecht et al. (2012) which showed that one sub-clade was only present in the most extreme habitat of the cold deserts in the Darwin Area (Antarctica). More investigations with much more extended taxon sampling needs to be done in order to decide which adaptations have occurred in response to extreme climatic conditions or particular ecological niches, and which speciation model applies.

Although no special ecological preferences are described in the literature for the genus *Asterochloris* (Peksa and Skaloud 2011), no representatives of this genus were found at the Tabernas desert in SE-Spain. *Asterochloris* species were, however, present at the more temperate and high alpine areas. There are at least two possible interpretations for these findings: Either the *Asterochloris* photobionts of *P. decipiens* cannot cope with the desert climate or the *P. decipiens* present at Tabernas preferentially selects other photobiont species. Attempting to answer this question is part of another study within the framework of the SCIN-project.

The highly variable occurrence of different photobiont types in association with the same mycobiont, *P. decipiens*, across all sampled habitats supports the opinion that flexibility in photobiont choice may influence the ecological amplitude of lichens (Peksa and Skaloud 2011). Low photobiont specificity is already known for several lichen species that show a wide ecological amplitude, e.g. *Lecanora rupicola*, and it appears that the key BSC lichen *P. decipiens* might employ a similar strategy for colonizing highly diverse habitats. In addition, the improved molecular techniques developed here can be important tools for future surveys of photobionts. Our results provide basic information that can underpin conservation measures to protect this highly specialized and diverse community of organisms that colonises and protects the soil surface in large areas of the world.

## Electronic supplementary material

Below is the link to the electronic supplementary material.

Online Resource 1: Lichen samples used in this study with information on collecting localities, voucher numbers, mycobionts, photobionts and other eukaryotic green micro algae (EGMA) including Genbank accession numbers. Several other specimens are included as well as the specimens directly collected at the four SCIN-investigation sites (DOC 215 kb)

Online Resource 2: Phylogeny of ITS sequences of *Trebouxia* specimens from the 4 SCIN-sites, together with samples from Antarctica, Austria and downloaded sequences from Genbank. The bars beside the phylogeny show the provenance of the specimens in the respective habitats. The bootstrap values with >70 support of MP and ML analyses were directly mapped on this Bayesian tree with >0.92 support (branches in bold) (EPS 1,554 kb)
