# Supplementary material for: High photobiont diversity in the common European soil crust lichen Psora decipiens
Source: Biodivers Conserv. 2014 Mar 8;23(7):1771–85. doi: 10.1007/s10531-014-0662-1 (PMC4058320; doi:10.1007/s10531-014-0662-1)
Supplement: Supplementary file 1 — Online Resource 1: Lichen samples used in this study with information on collecting localities, voucher numbers, mycobionts, photobionts and other eukaryotic green micro algae (EGMA) including Genbank accession numbers. Several other specimens are included as well as the specimens directly collected at the four SCIN-investigation sites (DOC 215 kb) [file 10531_2014_662_MOESM1_ESM.doc]

| Investigation sites | Herbarium ID | | Mycobiont | Photobiont | | | | | | | other green eukaryotic micro algae |
| --- | --- | --- | --- | --- | --- | --- | --- | --- | --- | --- | --- |
|  |  |  |  | *Trebouxia* sp. | | | *Asterochloris* sp. | | *Chloroidium saccharophilum* | |  |
|  |  |  |  | clade/species | ITS | *psb*J-L | clade/species | ITS | ITS | *psb*J-L | ITS |
| Tabernas/  Spain  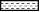 | T51457 | | *Psora decipiens* | *T*. sp. URa10 | KF907529 | KF907609 | - | - | - | - | - |
|  | T51458 | | *Psora decipiens* | *T*. sp. URa8 | KF907531 | KF907610 | - | - | - | - | - |
|  | T51459 | | *Squamarina lentigera* | *T*. sp. URa6 | KF907528 | KF907611 | - | - | - | - | - |
|  | T51460 | | *Psora decipiens* | *T*. sp. URa6 | KF907524 | KF907612 | - | - | - | - | - |
|  | T51461 | | *Psora decipiens* | *T*. sp. URa6 | KF907526 | KF907613 | - | - | - | - | - |
|  | T51462 | | *Psora decipiens* | *T*. sp. URa6 | KF907522 | KF907614 | - | - | - | - | - |
|  | T51463 | | *Psora decipiens* | *T*. sp. URa8 | KF907532 | KF907615 | - | - | - | - | - |
|  | T51464 | | *Psora decipiens* | *T*. sp. URa8 | KF907530 | KF907616 | - | - | - | - | - |
|  | T51465 | | *Psora decipiens* | *T*. sp. URa6 | KF907525 | KF907617 | - | - | - | - | - |
|  | T51466 | | *Psora decipiens* | *T*. sp. URa8 | KF907533 | KF907618 | - | - | - | - | - |
|  | Ps06/ALM-071* | | *Psora decipiens* | *T*. sp. URa6 | KF907523 | KF907580 | - | - | - | - | - |
|  | Ps07/ALM-073* | | *Psora decipiens* | *T*. sp. URa6 | KF907548 | KF907581 | - | - | - | - | - |
|  | Ps08/ALM-075* | | *Psora decipiens* | *T*. sp. URa6 | KF907549 | KF907582 | - | - | - | - | - |
|  | Ps09/ALM-077* | | *Psora decipiens* | *T*. sp. URa6 | KF907521 | KF907583 | - | - | - | - | - |
|  | Ps10/ALM-079* | | *Psora decipiens* | *T. asymmetrica* | KF907518 | KF907584 | - | - | - | - | - |
| Gynge Alvar/  Sweden  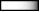 | T51467 | | *Psora decipiens* | - | - | - | *A*. sp. URa17 | KF907652 | - | - | KF907691 |
|  | T51468 | | *Psora decipiens* | *T*. *impressa* | KF907563 | KF907619 | *A*. sp. URa17 | KF907653 | - | - | KF907692 |
|  | T51469 | | *Psora decipiens* | *T*. sp. URa11 | KF907564 | KF907620 | *A*. sp. URa16 | KF907654 | - | - | - |
|  | T51470 | | *Psora decipiens* | - | - | - | *A*. sp. URa17 | KF907655 | - | - | - |
|  | T51471 | | *Psora decipiens* | *T*. sp. URa2 | KF907565 | KF907621 | *A*. sp. URa17 | KF907656 | - | - | - |
|  | T51473 | | *Psora decipiens* | - | - | - | - | - | - | KF907676 | - |
|  | Ps16/GYN-030* | | *Psora decipiens* | *T*. sp. URa12 | KF907552 | KF907589 | *A*. sp. URa17 | KF907647 | - | - | - |
|  | Ps17/GYN-056* | | *Psora decipiens* | - | - | - | *A*. sp. URa17 | KF907648 | - | - | - |
|  | Ps18/GYN-037* | | *Psora decipiens* | - | - | - | *A*. sp. URa17 | KF907649 | - | - | - |
|  | Ps19/GYN-051* | | *Psora decipiens* | *T. jamesii* | KF907553 | KF907590 | *A*. sp. URa17 | KF907650 | - | - | - |
|  | Ps20/GYN-006* | | *Psora decipiens* | *T*. sp. URa12 | KF907554 | KF907591 | *A*. sp. URa17 | KF907651 | - | - | - |
| Hochtor/  Austria  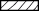 | T51474 | | *Psora decipiens* | *T*. sp. URa6 | KF907566 | KF907622 | *A*. sp. URa14 | KF907657 | - | KF907677 | KF907693 |
|  | T51475 | | *Psora decipiens* | *T*. *impressa* | KF907542 | KF907623 | - | - | - | KF907678 | - |
|  | T51476 | | *Psora decipiens* | - | - | - | - | - | - | KF907679 | KF907694 |
|  | T51477 | | *Fulgensia. bracteata* ssp. *deformis* | *T*. sp. URa4 | KF907509 | KF907624 | *A*. sp. URa15 | KF907658 | - | - | - |
|  | T51478 | | *Peltigera rufescens* | *T*. *impressa* | KF907540 | KF907625 | *A*. sp. URa14 | KF907659 | - | - | - |
|  | T51479 | | *Psora decipiens* | *T*. sp. URa11 | KF907567 | KF907626 | - | - | - | KF907680 | - |
|  | T51480 | | *Buellia elegans* | *T*. sp. URa2 | KF907568 | KF907627 | - | - | KF907672 | - | - |
|  | T51481 | | *Psora decipiens* | *T*. sp. URa6 | KF907569 | KF907628 | - | - | KF907673 | KF907681 | KF907695 |
|  | T51483 | | *Psora decipiens* | - | - | - | *A*. sp. URa14 | KF907660 | - | KF907682 | KF907696 |
|  | T51484 | | *F. bracteata* ssp. *d.* | *T*. sp. URa4 | KF907514 | KF907629 | - | - | - | - | - |
|  | T51485 | | *Buellia elegans* | *T*. *impressa* | KF907541 | KF907630 | - | - | - | - | - |
|  | T51486 | | *Psora decipiens* | *T*. sp. URa4 | KF907513 | KF907631 | - | - | - | KF907683 | - |
|  | T51487 | | *F. bracteata* ssp. *d.* | *T*. sp. URa4 | KF907515 | KF907632 | *A*. sp. URa15 | KF907661 | - | - | - |
|  | T51488 | | *Psora decipiens* | - | - | - | - | - | KF907674 | KF907684 | - |
|  | T51489 | | *Buellia elegans* | *T*. sp. URa11 | KF907538 | KF907633 | - | - | - |  | KF907697 |
|  | T51490 | | *Psora decipiens* | *T*. sp. URa11 | KF907539 | KF907634 | - | - | - | KF907685 | - |
|  | T51491 | | *F. bracteata* ssp. *d.* | *T*. sp. URa4 | KF907516 | KF907635 | - | - | - | - | - |
|  | T51492 | | *Buellia elegans* | *T*. sp. URa6 | KF907570 | KF907636 | - | - | KF907675 | - | - |
|  | Ps11/HOCH-076* | | *Psora decipiens* | *T*. sp. URa11 | KF907550 | KF907585 | - | - | - | - | - |
|  | Ps12/HOCH-042* | | *Psora decipiens* | *T*. sp. URa4 | KF907551 | KF907586 | - | - | - | - | - |
|  | Ps13/HOCH-038* | | *Psora decipiens* | *T*. sp. URa11 | KF907534 | KF907587 | - | - | - | - | - |
|  | Ps14/HOCH-034* | | *Psora decipiens* | *T*. sp. URa11 | KF907537 | KF907588 | - | - | - | - | - |
| Ruine Homburg/  Germany  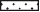 | T51493 | | *Fulgensia fulgens* | *T*. sp. URa6 | KF907527 | KF907637 | *A*. sp. URa16 | KF907662 | - | - | - |
|  | T51494 | | *Peltigera rufescens* | *T*. sp. URa6 | KF907571 | KF907638 | *A*. sp. URa16 | KF907663 | - | - | - |
|  | T51495 | | *Peltigera rufescens* | *T*. sp. URa6 | KF907520 | KF907639 | *A*. sp. URa16 | KF907664 | - | - | - |
|  | T51496 | | *Peltigera rufescens* | *T*. sp. URa6 | KF907572 | KF907640 | *A*. sp. URa16 | KF907665 | - | - | - |
|  | T51497 | | *Psora decipiens* | *T. asymmetrica* | KF907519 | KF907641 | *A*. sp. URa17 | KF907666 | - | - | - |
|  | T51498 | | *Psora decipiens* | *T*. *asymmetrica* | KF907517 | KF907642 | - | - | - | - | - |
|  | T51499 | | *Psora decipiens* | *T*. sp. URa6 | KF907573 | KF907643 | *A*. sp. URa17 | KF907667 | - | - | - |
|  | T51500 | | *Psora decipiens* | - | - | - | *A*. sp. URa17 | KF907668 | - | - | - |
|  | T51501 | | *Psora decipiens* | - | - | - | *A.magna* | KF907669 | - | - | KF907698 |
|  | T51502 | | *Psora decipiens* | *T*. *impressa* | KF907543 | - | *A*. sp. URa17 | KF907670 | - | - | KF907699 |
|  | T51503 | | *Psora decipiens* | *T*. sp. URa4 | KF907574 | KF907644 | *A*. sp. URa17 | KF907671 | - | - | KF907700  KF907701 |
|  | Ps01/GÖSS-001* | | *Psora decipiens* | - | - | - | *A*. sp. URa17 | KF907645 | - | - | KF907686 |
|  | Ps02/GÖSS-002* | | *Psora decipiens* | - | - | - | - | - | - | - | KF907687  KF907688 |
|  | Ps03/GÖSS-003* | | *Psora decipiens* | - | - | - | - | - | - | - | KF907689  KF907690 |
|  | Ps04/GÖSS-004* | | *Psora decipiens* | *T*. sp. URa9 | KF907546 | - | - | - | - | - | - |
|  | Ps05/GÖSS-005* | | *Psora decipiens* | *T*. sp. URa4 | KF907547 | KF907579 | *A*. sp. URa17 | KF907646 | - | - | - |
| Soil crust related lichens from other high alpine Areas | | | | | | | | | | | |
| Pasterze/A | | T34337 | *F. bracteata* ssp. *d.* | *T*. sp. URa4 | KF907510 | KF907594 | - | - | - | - | - |
| Hochtor/  Austria | | T38477 | *F. bracteata* | *T*. sp. URa4 | KF907511 | KF907595 | - | - | - | - | - |
|  |  | T38478 | *F. bracteata* | *T*. sp. URa4 | KF907512 | KF907596 | - | - | - | - | - |
|  |  | T40689 | *Buellia elegans* | *T*. sp. URa4 | KF907555 | KF907597 | - | - | - | - | - |
|  |  | T40700 | *Psora decipiens* | *T*. sp. URa13 | KF907556 | - | - | - | - | - | - |
| Seidenwinkeltal/A | | T46813 | *Peltigera rufescens* | *T*. sp. URa6 | KF907557 | KF907604 | - | - | - | - | - |
| Hinteres Modereck/  Austria | | T50556Pe | *Peltigera rufescens* | *T*. sp. URa13 | KF907558 | - | - | - | - | - | - |
|  |  | T50556T | *Tamnolia vermicularis* | *T. jamesii* | KF907559 | KF907605 | - | - | - | - | - |
|  |  | T50570C | *Cetraria muriacata* | *T*. sp. URa11 | KF907560 | KF907606 | - | - | - | - | - |
|  |  | T50570F | *F. bracteata* | *T*. sp. URa4 | KF907561 | KF907607 | - | - | - | - | - |
|  |  | T50570P | *Psora decipiens* | *T*. sp. URa4 | KF907562 | KF907608 | - | - | - | - | - |
| Saxicolous crustose lichens | | | | | | | | | | | |
| Antarctica | | T33612 | *Lecidella greenii* | *T*. sp. URa2 | JN204733 | KF907592 | - | - | - | - | - |
|  |  | T33712 | *Lecidea cancriformis* | *T*. sp. URa2 | JN204744 | KF907593 | - | - | - | - | - |
|  |  | T42992 | *L. cancriformis* | *T*. sp. URa1 | JN204771 | KF907598 | - | - | - | - | - |
|  |  | T42995 | *Lecidea andersonii* | *T*. sp. URa2 | JN204772 | KF907599 | - | - | - | - | - |
|  |  | T43003 | *L. andersonii* | *T*. sp. URa4 | JN204775 | KF907600 | - | - | - | - | - |
|  |  | T44634 | *L. cancriformis* | *T*. sp. URa1 | JN204801 | KF907601 | - | - | - | - | - |
|  |  | T44641 | *L. cancriformis* | *T*. sp. URa3 | JN204803 | KF907602 | - | - | - | - | - |
|  |  | T44692 | *L. cancriformis* | *T*. *jamesii* | JN204809 | KF907603 | - | - | - | - | - |
| Austria | | G1** | *Cultured Trebouxia* | *T*. sp. URa11 | KF907535 | KF907575 | - | - | - | - | - |
|  |  | G2** | *Cultured Trebouxia* | *T*. sp. URa6 | KF907544 | KF907576 | - | - | - | - | - |
|  |  | G3** | *Cultured Trebouxia* | *T*. sp. URa11 | KF907536 | KF907577 | - | - | - | - | - |
|  |  | G4** | *Cultured Trebouxia* | *T*. sp. URa6 | KF907545 | KF907578 | - | - | - | - | - |

T: Herbarium Türk (SZU)

*: Herbarium Stockholm (S)

**: Culture collection Georg Brunauer, University of Salzburg
